# Evaluation of Suboptimal Peak Inspiratory Flow in Patients with Stable COPD

**DOI:** 10.3390/jcm9123949

**Published:** 2020-12-05

**Authors:** Cristina Represas-Represas, Luz Aballe-Santos, Alberto Fernández-García, Ana Priegue-Carrera, José-Luis López-Campos, Almudena González-Montaos, Maribel Botana-Rial, Alberto Fernández-Villar

**Affiliations:** 1 Pneumology Department, Alvaro Cunqueiro University Hospital, 36312 Vigo, Spain; luz.aballe.santos@sergas.es (L.A.-S.); ana.priegue@iisgaliciasur.es (A.P.-C.); almudena.gonzalez.montaos@sergas.es (A.G.-M.); maria.isabel.botana.rial@sergas.es (M.B.-R.); alberto.fernandez.villar@sergas.es (A.F.-V.); 2 NeumoVigo I+i Research Group, Institute of Health Research Galicia Sur (IISGS, Instituto de Investigación Sanitaria Galicia Sur), 36312 Vigo, Spain; alberto.fernandez.garcia@outlook.es; 3 Medical-Surgical Unit of Respiratory Diseases, Institute of Biomedicine of Seville (IBiS), University Hospital Virgen del Rocío/University of Seville, 41013 Seville, Spain; lcampos@separ.es; 4 Centre for Biomedical Research in Respiratory Diseases Network (CIBERES), Institute of Health Carlos III, 28029 Madrid, Spain

**Keywords:** COPD, inspiratory flow, In-Check Dial G16, inhalation technique

## Abstract

Objective: Although the importance of assessing inspiratory flow in the selection of treatments for chronic obstructive pulmonary disease (COPD) is understood, evaluation of this factor is not yet widespread or standardized. The objective of the present work was to evaluate the peak inspiratory flow (PIF) of patients with COPD and to explore the variables associated with a suboptimal PIF. Methods: An observational, cross-sectional study was carried out at specialized nursing consultations over a period of 6 months. We collected clinical data as well as data on symptoms, treatment adherence, and patient satisfaction with their inhalers via questionnaires. PIF was determined using the In-Check Dial G16^®^ device (Clement Clarke International, Ltd., Harlow, UK). In each case, the PIF was considered suboptimal when it was off-target for any of the prescribed inhalers. The association with suboptimal PIF was evaluated using multivariate logistic regression and the results were expressed as the odds ratio (OR) with 95% confidence interval (CI). Results: A total of 122 COPD patients were included in this study, of whom 34 (27.9%) had suboptimal PIF. A total of 229 inhalers were tested, of which 186 (81.2%) were dry powder devices. The multivariate analysis found an association between suboptimal PIF and age (OR = 1.072; 95% CI (1.019, 1.128); *p* = 0.007) and forced vital capacity (OR = 0.961; 95% CI (0.933, 0.989); *p* = 0.006). Conclusions: About a third of patients in complex specialized COPD care have suboptimal PIFs, which is related to age and forced vital capacity.

## 1. Introduction

Inhaled therapy currently constitutes one of the cornerstones in the treatment of patients with airway diseases. The administration of drugs by inhalation allows direct access to the target organ which, compared to other administration routes, helps to achieve the same effects faster, at lower doses, and with fewer side effects, as a result of decreased systemic absorption [1].

To achieve an optimal therapeutic effect, patients must perform a correct inhalation technique. The use of an incorrect inhalation technique is one of the most frequent causes of therapeutic failure when inhalation devices (IDs) are used [2]. Carrying out inhalation correctly mainly depends on the patient completing all the steps described for performing this inhalation for each ID as well as avoiding critical errors [3]. In addition, performing a good inhalation technique with a dry powder devices not only involves correctly performing all the steps, but also requires the patient to generate a sufficient inspiratory flow to guarantee the release of the medication from the device into their airways and for the drug to be released from the excipient [4].

Evaluation of the inspiratory flow is especially relevant in patients with chronic obstructive pulmonary disease (COPD). It has been shown that the reduced inspiratory flow of these patients influences the effectiveness of the drugs being administered and this has relevant clinical consequences [5]. Thus, assessing the inspiratory flow of patients may be important in the choice of ID they should be prescribed [6]. Unfortunately, despite the relevance of this data, inspiratory flow is currently not often measured or standardized as part of the healthcare provided to patients with COPD, in part because comfortable, validated devices for this purpose are not routinely available.

Inspiratory flow measurement devices have recently been developed such as the Turbutest^®^ (Vitalograph, Maids Moreton, UK) [7], In-Check Dial^®^ [8], or new In-Check Dial G16^®^ model (Clement Clarke International, Ltd., Harlow, UK) [9], which evaluate the PIF of patients and, in the latter case, can simulate the internal resistance of the prescribed ID. The use of these devices could help researchers explore the inspiratory capacity of patients with COPD and can therefore serve as a guide in the personalization of the treatment of these patients. However, the clinical characteristics associated with having an adequate inspiratory flow have not yet been sufficiently assessed. Thus, the objective of this work was to evaluate the inspiratory flow of patients with COPD by using the In-Check Dial G16^®^ device, and to explore the variables associated with suboptimal inspiratory flow. These results will help clinical decision-making when choosing the best type of ID to prescribe to patients.

## 2. Methods

This was an observational, cross-sectional study in which information about patients with COPD was prospectively and consecutively collected over a period of 6 months and analyzed in specialized nursing consultations in a tertiary hospital. All the patients included had a confirmed diagnosis of COPD according to current recommendations [10], had attended the high-resolution nursing consultation (specialized consultation, where the nurse conducts therapeutic education, and administers tests such as spirometry, etc.) or individual consultation with a case manager nurse for highly complex COPD (a nurse who provides additional education, care coordination and telephone attention on demand for these patients), were in a stable disease phase (defined as not experiencing any flare-ups in at least the 3 previous months), and had been prescribed an ID for at least 6 months, excluding nebulized therapy at home. Patients who refused to participate in the study, who were unable to collaborate in the PIF measurement technique due to physical or mental conditions or who were experiencing a COPD exacerbation at the time of the visit were excluded.

### 2.1. Process

After recruiting the patients to participate in this study, clinical data related to their COPD was collected on the day of their visit using a standardized questionnaire. This questionnaire included epidemiological data (age, sex, and educational level), the patient’s smoking status and cumulative consumption, and clinical data, including the degree of dyspnea according to the modified scale from the Medical Research Council (mMRC), the number of exacerbations in the last year, and the number of IDs prescribed as maintenance treatment for COPD. Based on these data, the patients were classified according to the 2019 Global Initiative for Obstructive Lung Disease (GOLD) document [11].

Next, various questionnaires were administered to assess the impact of COPD: the COPD Assessment Test (CAT) questionnaire was used in its validated Galician version [12], adherence to inhaled treatment was measured using the Inhaler Adherence Test (TAI) questionnaire [13], and for assessment of inhaler satisfaction and preference, the Feeling of Satisfaction with Inhaler 10 questionnaire (FSI-10) [14] was used.

Subsequently, we assessed the inhalation technique and recorded any errors the patients made when using their usual IDs. In the case of patients with more than one prescribed ID, their technique was considered completely correct when the patient did not make any critical errors with any of their prescribed devices. Subsequently, we measured the PIF reached by the patient according to the devices they had been prescribed, using the In-Check Dial G16^®^. Finally, the spirometry data from the bronchodilator test, performed according to current standards of practice [15], were collected: values of forced vital capacity (FVC), forced expiratory volume in 1 s (FEV_1_) and its ratio. The body mass index (BMI) was also calculated.

### 2.2. Measurement Tools

The education level of the patients was classified as a qualitative variable into three categories: primary education, secondary education or university-level education. Smoking history was quantified according to the accumulated consumption in pack-years [16].

The TAI questionnaire consists of two complementary questionnaires. The first comprises 10 items designed to measure therapeutic adherence and its intensity (good, intermediate, or bad), and can be completed by the patient. Each item is scored from 1 to 5 to obtain an overall score between 10 and 50, where higher scores indicate better adherence. The second consists of 2 added items which must be completed by healthcare personnel and are designed to evaluate the pattern of non-compliance by scoring 1 or 2 points in each case, where higher scores indicated better adherence. Additionally, the 12-item TAI also allows the type of non-compliance (erratic, deliberate, or unconscious) to be identified.

The FSI-10 questionnaire is a self-administered tool for assessing the preferences and satisfaction of patients regarding their inhalation devices. It comprises 10 questions, which include items related to comfort, difficulty, portability and use of the device. Each question has 5 response options on a 5-point Likert scale (‘a lot’, ‘quite a lot’, ‘somewhat’, ‘a little’ and ‘very little’), scored from 5 to 1. The overall possible score for the FSI-10 is 50 points, where higher scores indicate a lower degree of satisfaction [14].

PIF measurement was performed with the In-Check Dial G16^®^ device for each of the IDs that the patient had been prescribed. This device can measure the PIF for up to 16 different IDs (including all the devices on the market at the time of this study), by simulating their internal resistance and expressing these values in L/min with a measurement range of 15–120 L/min (±10 L/min) [9]. The In-Check Dial G16^®^ PIF estimates are based on comparable internal measurements performed under standard conditions by the developers [9] and allows physicians to evaluate whether a given patient will be able to achieve the estimated PIF for each ID they have been prescribed, based on their individual resistance level.

The device categorizes dry powder inhalers (DPIs) according to their internal resistance as follows: low resistance (R1: Breezhaler^®^), medium-low resistance (R2: Accuhaler^®^, Diskhaler^®^, and Ellipta^®^), medium resistance (R3: Genuair^®^, Spiromax^®^, Clickhaler^®^, and Turbuhaler S^®^), medium-high resistance (R4: Turbuhaler^®^, Twisthaler^®^, Nexthaler^®^, and Easyhaler C^®^) and high resistance (R5: Easyhaler M^®^ and Handihaler^®^). To evaluate each ID, the mouthpiece is placed according to the ID being tested (according to a color code marked on the meter’s technical sheet), and the most suitable orifice caliber is selected to simulate the internal resistance of the ID.

These values are determined for DPIs, whereas no resistance is provided for pressurized metered-dose inhalers (pMDIs) and slow-mist inhalers (SMIs). Once the inhaler to be tested had been selected and the In-Check Dial G16^®^ device set-up accordingly, the patient inhaled through a mouthpiece, simulating their normal inhalations through the ID.

Although the PIFs considered adequate for each type of ID vary according to the source consulted, for this study we used the PIFs proposed by Mahler et al. [6] at 30–60 L/min for pMDIs, ≤30 L/min for pMDIs with chamber, 15–50 L/min for SMIs, 60 L/min for low and medium resistance DPIs (R1 to R4), and 30 L/min for high resistance DPIs (R5). For patients with more than one prescribed ID, their PIF was considered adequate when their inhalation capacity fell within the ranges for all their prescribed inhalers.

### 2.3. Ethical Factors

The patient data database was anonymized, and no data were collected that could be used to identify the patient. The data were obtained, treated, conserved, communicated and transferred in accordance with the provisions of the rules and regulations in force in Europe. This study was approved by the Research Ethics Committee of Galicia, with file reference number 2017/120. All the patients included in this work gave their informed consent in writing to indicate that they agreed to participate.

### 2.4. Statistical Analysis

Data analysis was performed with SPSS software (version 26.0, IBM Corporation, Armonk, NY, USA), setting the alpha error at 0.05. We performed a descriptive statistical analysis of the main study variables, representing the qualitative variables as frequencies and percentages and the quantitative variables as means and standard deviations when they fit a normal distribution curve, or otherwise, as medians and interquartile ranges (IQRs).

We initially studied the possible demographic, clinical, and functional factors associated with not achieving an adequate PIF individually with unadjusted bivariate analyses, using Chi-squared tests for the categorical variables and Student *t* tests for the independent data for quantitative variables. Likewise, a bivariate approach was used to analyze the association of the adherence, inhalation technique, satisfaction with the inhaler and clinical event (such as exacerbations or the clinical impact of the disease) variables with an adequate PIF. Subsequently, a conditional forward multivariate binomial logistic regression analysis was performed which included all the variables with a significance of *p* < 0.1 in the bivariate analysis. The results were expressed as odds ratios (OR) with an estimation of the 95% confidence intervals (CIs).

## 3. Results

A total of 122 patients were included during the 6 months of this study, of whom 88 (72.1%) achieved an adequate PIF for all their IDs and 34 (27.9%) presented a suboptimal PIF for at least one of their inhalers. The main clinical characteristics of the patients in this study cohort are shown in Table 1. Around 64% (78) of the patients had only completed primary education, 29% (35) had completed secondary education, and 7% (9) had university-level degrees. All the patients reported a history of smoking, and 40.2% of them were active smokers. According to the 2019 GOLD classification, 12.4% of the patients belonged to group A, 25.6% to group B, 13.2% to group C and the remaining 48.8% to group D.

A total of 229 IDs were tested, of which 43 (18.7%) were pMDIs or SMIs, 49 (21.4%) were R1 resistance DPIs, 33 (14.4%) R2 resistance, 17 (7.4%) R3 resistance, 65 (28.4%) R4 resistance and 22 (9.6%) R5 resistance. According to our inclusion criteria, all the patients had been using their inhalers for at least 6 months and 103 (84.4%) of them had been using their IDs for more than 1 year.

In the per-device analysis, all the patients with pMDIs or SMIs achieved an adequate PIF, while the PIF was suboptimal in 42 patients (22.6% of DPIs). Table 2 shows the number of cases with suboptimal PIFs as a function of the resistance of the dry powder ID used as well as the average PIF in each group. A higher percentage of suboptimal PIF was observed in patients using R4 DPIs compared to the other types of DPI (*p* < 0.01). Significant differences (*p* < 0.01) were also found between the mean PIFs of each group of DPI as a function of resistance, except between R2 and R3 (*p* = 0.73).

Table 1; Table 3 show the bivariate analyzes between the PIF groups, comparing different variables related to the characteristics of the participants, clinical presentation, adherence, technique, and satisfaction with the ID. The multivariate analysis found an independent and statistically significant association with a suboptimal PIF for age (OR = 1.072; 95% CI (1.019, 1.128); *p* = 0.007) and FVC in ml (OR = 0.961; 95% CI (0.933, 0.989); *p* = 0.006).

## 4. Discussion

The results of the present work, in which we consecutively assessed the PIF of patients with COPD attending a nursing consultation, showed that: (1) almost a third of the patients with stable COPD who went for a follow-up in specialized pulmonology consultations did not reach the optimal PIF for the inhalers they had been prescribed; and (2) the main variables associated with suboptimal PIF were age (which showed a direct relationship) and FVC (which was inversely related). The relationship between these variables and PIF revealed an association that physicians could consider in the future when developing algorithms for clinical decision-making for use with patients with COPD.

It is well known that correct inhalation technique is one of the cornerstones underpinning the success of inhaled therapy. However, selecting the ideal ID for each patient still remains a challenge for clinicians [17]. Inspiratory flow has been reported to be causally related to the effectiveness of inhalation through dry powder devices. In this context, the inhalation technique must be able to generate sufficient inspiratory flow to guarantee both the release of the medication from the ID as well as its adequate disintegration, resulting in the separation of the drug from the lactose excipient [18]. It would therefore be especially useful if a simple method to allow inspiratory flow to be assessed in daily clinical practice were available.

One of the main strengths of this work was the fact that we used the In-Check Dial G16^®^ device because, as demonstrated elsewhere [19], it allows every dry powder ID to be tested based on its internal resistance, with reproducible measurements at different times of day. We evaluated all the devices used by the patients included in our cohort without limiting ourselves to specific devices, as in previous studies. Furthermore, we assessed the devices that the patients in our study population had been prescribed in real clinical practice at the time of their inclusion, without giving any educational advice on the management of these IDs beforehand. Finally, this is the only study available in the literature to date in which the analysis combined sociodemographic, clinical and functional factors, as well as data related to the inhalation technique, therapeutic adherence and patient satisfaction with the device.

However, a number of methodological considerations must also be taken into account in order to correctly evaluate our results. First, although the sample included patients with different degrees of functional severity, we only included patients from a single center, and so similar analyses should be carried out to reproduce these results in other cohorts. Second, it is worth mentioning that the number of women included in our population was low. This was because, although we recruited the patients consecutively, COPD is far more prevalent in men than in women in our area [20], resulting in the latter being underrepresented. However, we know that COPD has a different impact on women and that the male/female gap affects various areas of the disease, including inhaled therapies [21,22,23]. Therefore, in future studies, it would be advisable to try to reproduce our results in a cohort which includes a higher proportion of women than in this current work. Another limitation is that only data from forced spirometry of the patients were recorded: lung volumes were not recorded, in order to detect restrictive pathologies. In addition, there are no data on comorbidity or frailty, so this aspect could not be analyzed, although all the patients were independent and in good general health.

Previous studies have also reported high prevalence of suboptimal PIF in COPD patients, ranging from 20% to 78% in the populations studied [19,24,25,26]. These frequencies may vary depending on the population studied, the device used to assess PIF, the IDs assessed and the clinical context, because some of these previous studies were carried out during periods of hospitalization [25]. In the present work, we used the most advanced PIF assessment device available to date to consecutively assess all the IDs used by the COPD patients in our cohort, regardless of their degree of functional severity. Therefore, this work contributes new, valuable data to our global understanding of PIF in our community.

Numerous factors have been associated with inspiratory flow in previous studies, including female sex, age, short stature, internal ID resistance, inhalation effort and lung function [19,25,27,28]. In the present work, we found that age and FVC were the main variables that were independently associated with suboptimal PIF. The association between age and PIF remains controversial. Some works published in the literature have found this association, [5,25,28,29,30], while other authors have been unable to confirm these findings [19,26,31]. These discrepancies may be because, in reality, in these studies, age acts as a surrogate variable for other more important factors such as the presence of comorbidities or the patient’s state of frailty [32]. The relationship between the aging process and the ability to generate a sufficient PIF is complex and should be explored further.

Regarding the association between suboptimal PIF and FVC, it is worth noting that FEV_1_ was omitted from the multivariate model in our functional assessment. This finding is consistent with the results reported by other authors [24,29]. In contrast, a recent study which also determined PIF using the In-Check Dial G16^®^ in a cohort of 138 patients with stable COPD found an association with both FEV_1_ and FVC [33]. One of the few studies that included patients with severe COPD also observed a statistically significant correlation between FEV_1_ and PIF, but in this case, only for the Ellipta^®^ ID [34].

However, it is important to remember that COPD is a disease which predominantly limits expiratory airflow and in which inspiratory flows are preserved until the very advanced stages of the disease. Therefore, we would expect inspiratory flow would only be affected in patients with very severe functional impairment. Thus, given that FVC reflects the volume of air that a patient can voluntarily mobilize, a factor related to the inspiratory flow they can generate, we might expect this metric to remain relatively stable in these patients.

Notably, the PIF is often affected in cases of very severe obstruction, probably due to air trapping and a reduction in secondary FVC. Specifically, Duarte et al. found a significant correlation between PIF and the severity of air trapping, as represented by the ratio of residual volume and total lung capacity [31]. Finally, patients with very severe COPD and hyperinflation frequently present an association between the appearance of different degrees of malnutrition and sarcopenia [35], which can condition the strength of the inspiratory musculature and, therefore, the PIF generated.

Another aspect, the relationship between inhalation technique and the type of therapeutic non-compliance with suboptimal PIF, has not been analyzed in any other study, so it is not possible to compare the results. It could be a matter of discussion whether suboptimal PIF is a cause or consequence of incorrect inhalation technique. Since, in our work, the review of inhalation practice was carried out in some patients before and in others after the PIF measurement, a new line of investigation could be whether the instruction and correction of errors in previous inhalation practice could lead to changes in the PIF.

In this context, nursing consultations may represent useful opportunities to improve disease control in patients with COPD. Once a treatment has been prescribed, it is often nurses who care for patients in the long term, and they may even become the main providers of day-to-day clinical care for these patients, helping them to resolve problems such as the management of their IDs. This places nurses in a key position to monitor inhaler technique, communicate with the patient, improve adherence and even suggest alternative treatments if the proposed therapy is incompatible with the patient [36].

In conclusion, in the era of personalized medicine, a wide range of IDs are available on the market, and it is important to select the one that best suits the characteristics of each patient, considering their preferences and even their lifestyles. The evaluation of PIF is a key step in selecting a suitable ID that will optimize patient inhalation technique and ensure the deposition of particles in their airways. This measurement should therefore be carried out in every patient with COPD before prescribing an ID because of its implications in the control of this disease [37]. Thus, in conclusion, the In Check Dial G16^®^ may be a useful tool for selecting the ID that should be prescribed to each patient. Based on our findings, assessment of the PIF should be prioritized in older patients with decreased FVCs.

## Figures and Tables

**Table 1:** Bivariate analysis of the sociodemographic, clinical, and functional variables that may predict suboptimal peak inspiratory flow.

	Total (*n* = 122)	Adequate PIF (*n* = 88)	Sub-Optimal PIF (*n* = 34)	*p* Value *
Males	106 (87%)	76 (86%)	30 (88%)	0.99
Age (years)	68 ± 10	67 ± 10	72 ± 10	0.09
Primary education	78 (64%)	53 (60%)	25 (74%)	0.17
Active smoking	49 (40%)	34 (39%)	15 (44%)	0.58
BMI (Kg/m^2^)	28.4 ± 5.6	28.4 ± 5.4	28.5 ± 6.1	0.9
Forced expiratory volume in 1 s (FEV_1_) (mL)	1350 ± 528	1387 ± 562	1129 ± 371	0.04
FEV1 (%)	47 ± 16	49 ± 17	43 ± 14	0.08
Forced vital capacity (FVC) (mL)	2852 ± 908	3005 ± 982	2456 ± 500	<0.01
FVC (%)	77 ± 18	80 ± 19	70 ± 12	<0.01

Values are expressed as the mean ± standard deviation or absolute (relative) frequencies according to the nature of the variable. * Calculated using Chi-squared tests for categorical variables and Student *t* tests for quantitative variables with independent data. BMI: body mass index. FEV_1_: Forced expiratory volume in 1 s. FVC: forced vital capacity.

**Table 2:** Analysis of the mean PIF in patients with suboptimal PIF who used dry powder inhalers (DPI) as a function of the degree of resistance.

Resistance *	Number of Patients	PIF (L/min)	Sub-Optimal PIF
R1	49	95 (21)	5 (10.2%)
R2	33	74 (18)	6 (18.1%)
R3	17	74 (19)	2 (11.7%)
R4	65	62 (14)	26 (40%)
R5	22	40 (12)	3 (13.6%)

Values are expressed as the mean ± standard deviation or absolute (relative) frequencies according to the nature of the variable. * The values are defined in the text. PIF: peak inspiratory flow.

**Table 3:** Comparative analysis of the inhaled therapy variables, exacerbations and clinical impact in the groups with adequate or suboptimal PIF.

	Total (*n* = 122)	Adequate PIF (*n* = 88)	Sub-Optimal PIF (*n* = 34)	*p* Value *
Some critical error in inhalation technique	73 (60%)	47 (53%)	26 (77%)	0.02
Intermediate or bad adherence (TAI)	32 (26%)	27 (31%)	5 (15%)	0.07
Erratic non-compliance	21 (17%)	19 (22%)	2 (6%)	0.04
Deliberate non-compliance	11 (9%)	11 (13%)	0 (0%)	0.03
Unconscious non-compliance	74 (61%)	47 (53%)	27 (79%)	<0.01
FSI-10 (score)	44.7 ± 5.8	45.3 ± 5.6	43.5 ± 6.1	0.13
Medium/low satisfaction (FSI-10)	25 (21%)	17 (19%)	9 (27%)	0.39
Number of exacerbations in last year	1.2 ± 1.4	1.06 ± 1.3	1.56 ± 1.3	0.06
≥2 exacerbations in last year	38 (31%)	22 (25%)	16 (47%)	0.02
No exacerbations in last year	46 (38%)	37 (42%)	9 (27%)	0.11
Dyspnea score (mMRC)	1.6 ± 1	1.63 ± 1	1.73 ± 1.1	0.63
3–4 mMRC dyspnea score	20 (16%)	14 (16%)	6 (18%)	0.81
CAT score	15 ± 7	15 ± 8	15 ± 6	0.99

Values are expressed as the mean ± standard deviation or absolute (relative) frequencies according to the nature of the variable. * Calculated using Chi-squared tests for categorical variables and Student *t* tests for quantitative variables with independent data. TAI: Inhaler Adherence Test questionnaire. FSI-10: Feeling of Satisfaction with Inhaler 10 questionnaire. mMRC: modified Medical Research Council. CAT: COPD Assessment Test.
